# Prescription stimulants in individuals with and without attention deficit hyperactivity disorder: misuse, cognitive impact, and adverse effects

**DOI:** 10.1002/brb3.78

**Published:** 2012-07-23

**Authors:** Shaheen E Lakhan, Annette Kirchgessner

**Affiliations:** Global Neuroscience Initiative FoundationLos Angeles, California

**Keywords:** Amphetamine, athletes, attention deficit hyperactivity disorder, cognition, methylphenidate, misuse, performance, students

## Abstract

Prescription stimulants are often used to treat attention deficit hyperactivity disorder (ADHD). Drugs like methylphenidate (Ritalin, Concerta), dextroamphetamine (Dexedrine), and dextroamphetamine-amphetamine (Adderall) help people with ADHD feel more focused. However, misuse of stimulants by ADHD and nonaffected individuals has dramatically increased over recent years based on students' misconceptions or simple lack of knowledge of associated risks. In this review, we discuss recent advances in the use and increasing misuse of prescription stimulants among high school and college students and athletes. Given the widespread belief that stimulants enhance performance, there are in fact only a few studies reporting the cognitive enhancing effects of stimulants in ADHD and nonaffected individuals. Student athletes should be apprised of the very serious consequences that can emerge when stimulants are used to improve sports performance. Moreover, misuse of stimulants is associated with dangers including psychosis, myocardial infarction, cardiomyopathy, and even sudden death. As ADHD medications are prescribed for long-term treatment, there is a need for long-term safety studies and education on the health risks associated with misuse is imperative.

## Introduction

Attention deficit hyperactivity disorder (ADHD) is a treatable neurobehavioral disorder that is defined by persistent and maladaptive symptoms of hyperactivity/impulsivity and inattention (American Psychiatric Association 2000). ADHD is one of the most common psychiatric conditions of childhood (Wilens et al. 2002). Based on the Heath Resources and Services Administration's National Survey of Children's Health, the percentage of children aged 4–17 years diagnosed with ADHD increased from 7.8% in 2003 to 9.5% in 2007, representing a 21.8% increase in just 4 years (Centers for Disease Control and Prevention 2010). ADHD is diagnosed in boys at a rate of two to four times that of girls, although this observation may be the result of referral patterns from teachers (Sciutto et al. 2004; Kutcher 2011). Although ADHD was once regarded as a disorder of childhood and adolescence, an estimated 50% of patients diagnosed with ADHD under the age of 18 years continue to have symptoms as an adult (Wilens et al. 2004). Overall, the prevalence of ADHD in adults ranges from 3.5% to 4.5% (Kessler et al. 2006). Differences across ethnic groups within the United States are sometimes found, but seem to be more of the function of social class than ethnicity (Bloom and Cohen 2007). ADHD is found in all countries surveyed with rates similar to, if not higher than, those found in North America (Faraone et al. 2007; Polanczyk et al. 2007). Thus, adult ADHD is one of the most common adult psychiatric disorders.

Individuals with ADHD often have substantial functional impairment in academic, family, and social settings. Youth with ADHD are at an increased risk for academic failure because of learning or language problems. Other consequences associated with ADHD include dangerous driving, impaired peer relationships, delinquent behavior, and impulsive sexuality (Putukian et al. 2011; Visser et al. 2012). Moreover, when ADHD is untreated, there is increased prevalence of certain psychological disorders (e.g., major depression, bipolar disorder, conduct disorder, oppositional-defiant disorder, antisocial personality, substance use, and anxiety) (Faraone et al. 1997; Rasmussen and Gillberg 2000; Kollins et al. 2005; Biederman et al. 2006). However, early treatment may decrease negative outcomes of ADHD including the rate of conduct disorder and adult antisocial personality disorder (Dopheide and Pliszka 2009).

There are both pharmacological and nonpharmacological (e.g., cognitive behavioral therapy [CBT]) treatments of ADHD. Stimulants, such as methylphenidate (MPH; Ritalin and Concerta) and dextroamphetamine-AMP (d-AMP; Adderall) are the most common pharmacologic treatments (The MTA Cooperative Group 1999) and abundant data support the potentially positive effects of prescription stimulants for the majority of children, adolescents, and adults with ADHD. Experts estimate that approximately 60% of children with ADHD are treated with prescription stimulants (Center for Disease Control and Prevention 2005a); therefore, approximately three million children in this country take stimulants for problems with focusing. At the same time, many studies have revealed the numerous adverse effects associated with prescription stimulants when they are used inappropriately.

Stimulants are classified as Schedule II drugs (i.e., providing positive medicinal effects but also considerable abuse potential). The nonmedical use of prescription stimulants represents the second common most form of illicit drug use in college, second only to marijuana use (Johnston et al. 2004). Indeed, many consider stimulants – whether obtained by prescription or illicitly – a convenient option to improve performance or to induce euphoria (get “high”). Major daily newspapers such as *The New York Times* have reported a trend toward growing use of prescription stimulants, commonly called “smart pills,” by high school and college students for enhancing school or work performance (Jacobs 2005). Unfortunately, media reports appear to condone this behavior as 95% of articles mentioned at least one possible benefit of using a prescription stimulant for neuroenhancement, but only 58% mentioned any risks/side effects (Partridge et al. 2011). Stimulant misuse is often predicted on individuals' misconceptions or simple lack of knowledge of associated risks.

This review discusses recent studies regarding the use and misuse of stimulants among high school and college students, including athletes, with and without ADHD. Given the widespread belief that prescription stimulants are “smart pills,” we address if these drugs actually enhance cognition in a healthy individual. Athletes may see stimulants as a way to help maintain physical fitness for their competitive sport or to improve their concentration. Finally, we elaborate on the long-term effects of chronic stimulant use. Addiction and tolerance are major concerns, as are psychosis and cardiovascular effects. Surprisingly, these associated risks of stimulant misuse are not frequently addressed in the media and literature. Clearly, the widespread misuse of prescription stimulants represents an important public health issue faced by students, school officials, health centers, and parents.

## Methods

This review was initiated with a PubMed search of the US National Library of Medicine with combinations of the following key words: “Adderall,” “amphetamine,” “methylphenidate,” “dexamphetamine,” “ADHD,” “misuse,” “illicit use,” “non-prescription use,” “non-medical use,” “diversion,” “students,” and “athletes.” A review of all titles was conducted to include only pertinent publications. A hand search of psychiatry journals was performed and reference lists from relevant studies were searched.

## Prescription stimulant use in ADHD

It is estimated that about two-thirds of the children diagnosed with ADHD receive pharmacological treatment (Centers for Disease Control and Prevention 2010) and the majority of medications used are stimulants (Center for Disease Control and Prevention 2005b). The prescribed use of stimulant medications to treat ADHD in children age 18 and younger rose steadily from 1996 to 2008, from an estimated 2.4% in 1996 to an estimated 3.5% of US children in 2008 (Zuvekas and Vitiello 2011). Overall, prescription stimulant use among 6- to 12-year-olds is highest, going from 4.2% in 1996 to 5.1% in 2008; however, the fastest growth rate occurred among 13–18 year olds, going from 2.3% in 1996 to 4% in 2008. Prescription stimulant use remained consistently low in the West than in other US regions and in lower racial/ethnic minorities.

MPH and d-AMP are the most widely used prescription stimulants approved by the US Food and Drug Administration (FDA) for the treatment of ADHD. MPH is a short-acting stimulant drug. Generic MPH is available in many forms, and several versions of the long-acting MPH have been introduced, with Concerta getting the largest share of the market. According to the U.S. Drug Enforcement Administration (DEA), MPH has been the fourth most prescribed controlled substance in the United States since 2003, with over 58,000 Americans purchasing MPH in 2006 (Department of Justice: Drug Enforcement Administration 2008). Both the production and prescription of MPH has risen as the diagnosis of ADHD has concurrently increased. In addition, with the realization that ADHD is a lifelong disorder, MPH has become more commonly prescribed for adolescents and adults, and treatment duration has increased (Horrigan 2001). Both MPH and d-AMP are efficacious and well-tolerated medications and remain the first choice for short duration management in adolescent and adult ADHD (Faraone and Glatt 2010). Although the precise mechanisms underlying the action of these medications are not completely understood, they appear to increase the availability of dopamine, which could account for their therapeutic effects.

Although ADHD is a multifactorial disorder, disrupted dopamine (DA) neurotransmission plays an important role in its pathophysiology. In addition, polymorphisms in the dopamine D1 receptor (DRD1) are associated with the disorder (Misener et al. 2004). MPH and d-AMP both enhance DA signaling in the brain. MPH increases DA by blocking dopamine transporters (DATs) and AMP by releasing DA from the nerve terminal using the DAT as carrier (Kuczenski and Segal 1997). In healthy controls and in adolescents and adults with ADHD (Rosa-Neto et al. 2005; Volkow et al. 2007), MPH significantly increased DA in the ventral striatum (VS) (Volkow et al. 2012), a crucial brain region involved with motivation and reward (Wise 2002). Moreover, intravenous MPH-induced increases in DA in the VS were correlated with improvement in symptoms of inattention after long-term oral MPH treatment. Historically, the core feature of ADHD has been characterized as one of attention deficit, but increasing evidence suggests that a reward and motivation deficit may be of equal importance. It has been proposed that increasing DA in the VS would enhance the saliency of the task, thus improving attention in ADHD (Volkow et al. 2012). Intravenous MPH also significantly increased DA in the prefrontal and temporal cortices that were associated with decreased ratings of inattention, which may be therapeutically relevant.

The widespread use of prescription stimulants for ADHD has not been without critics. In recent months, we have heard speculation about whether ADHD is a real disease, and if it is real, whether it is being grossly over-diagnosed. Disorders often become widely diagnosed after drugs come along that can alter a set of suboptimal behaviors. In this way, Ritalin and Adderall helped make ADHD a household name. If there is a pill that can clear up the wavering focus of sleep-deprived youth, then those rather ordinary states may come to be seen as syndrome. A recent opinion piece entitled “Ritalin Gone Wrong” in the *New York Times* (Sroufe 2012) by psychology professor L. Alan Sroufe argues that attention-deficit drugs do more harm than good over the long term, a conclusion other professionals in his field dispute. Studies have shown that children who take MPH can show reductions in ADHD symptomatology (inattention, hyperactivity, and impulsivity) and gains in social and classroom behaviors. Studies of adults with ADHD have confirmed its usefulness for this population as well. However, the benefits of prescription stimulants on ADHD symptomatology do not appear to last long.

The Multimodal Treatment Study of Children with ADHD (MTA) compared four distinct treatment strategies during childhood for children diagnosed with DSM-IV ADHD, Combined Type (The MTA Cooperative Group 1999). Children were randomly assigned to 14 months of (a) systematic medication management (MedMgt), which was initial placebo-controlled titration, three times a day dosing, 7 days a week, and monthly 30-min clinic visits, (b) multicomponent behavior therapy (Beh), which included 27-session group parent training supplemented with eight individual parent sessions, an 8-week summer treatment program, 12 weeks of classroom administered behavior therapy with a half-time aide, and ten teacher consultation sessions, (c) their combination (Comb), or (d) usual community care (CC). This randomized, six-site, controlled clinical trial featured rigorous diagnostic criteria at study entry and compared the relative effectiveness of treatments of well-established efficacy. The initial MTA findings reported that all groups showed improvement over baseline at the end of the 14-month treatment period; however, the Comb and MedMgt group participants showed significantly greater improvements in ADHD symptoms than did the Beh or CC participants. By the next follow-up, 3 years after enrollment, there were no longer significant treatment group differences in ADHD symptoms or functioning (Jensen et al. 2007). Molina et al. (Molina et al. 2009) reported the next two follow-up assessments of the MTA sample at 6 and 8 years after random assignment, when the sample ranged in age from 13 to 18 years and found similar findings.

## Prevalence of prescription stimulant misuse

The misuse of a stimulant medication – taking a stimulant not prescribed by a physician or in a manner not in accordance with physician guidance – has been growing over the past two decades. In fact, in the past 10 years there has been a surge in prevalence rates of nonprescription stimulant use among both adolescents and young adults. In general, nonprescription use of MPH in 2000 was reported as 1.2% and in 2006 this number had risen to 2%. Breaking the sample down by age, nonprescription use among adolescents (ages 12–17) went from 2.2% to 1.8% between 2000 and 2006, a slight decrease. Among college-aged individuals (ages 18–25), however, usage increased significantly from 3.6% in 2000 to 5.4% by 2006. Finally, among those 26 and older, usage is the lowest of any group, but rates are rising. In 2000, only 0.7% reported any lifetime usage of MPH, but this number had doubled to 1.5% by 2006 (Bogle and Smith 2009).

The majority of research on the misuse of prescription stimulants has focused on undergraduate college students. The nonprescription use of stimulants has increased in this population, to the extent that the misuse of prescription stimulants is second only to marijuana as the most common form of illicit drug use among college students (Johnston et al. 2004). A 2001 nationwide self-reported survey of more than 10,000 students from 4-year universities in the United States reported a 6.9% lifetime prevalence of nonprescription stimulant misuse, including a past-year prevalence of 4.1% and a past-month prevalence of 2.1% (McCabe et al. 2005). Colleges with the highest past-year prevalence rates were typically located in the northeastern United States, which is corroborated by other reports (McCabe et al. 2005). A study by Teter et al. (2005) of 9161 undergraduates reported an 8.1% lifetime nonprescription stimulant misuse rate among college students, including 5.4% over the past year. According to a 2002 survey of a single US college, 35.5% of undergraduates reported using stimulants without a prescription, with greater frequency occurring in males compared with females (Low and Gendaszek 2002).

The majority of nonprescription stimulant users reported obtaining the drugs from a peer with a prescription – a process termed diversion. The diversion of stimulants is very common and can begin in childhood, adolescence, or young adulthood. A study conducted by Wilens et al. (2008) reported that lifetime rates of diversion ranged from 16% to 29% of students with stimulant prescriptions asked to give, sell, or trade their medications (Wilens et al. 2008). One survey reported that 23.3% of middle and high school students taking prescribed stimulants had been solicited to divert their medication to others at a rate that increased from middle school to high school (McCabe et al. 2004). A review of 161 elementary and high school students prescribed the stimulant MPH revealed that they had been asked to give or sell their medication to others (Musser et al. 1998). Data has shown that the diversion continues among college students. McCabe et al. found 54% of college students who were prescribed stimulants for ADHD had been approached to divert their medication (McCabe and Boyd 2005). Nearly 29% of 334 college students had sold or given their medication to others (Upadhyaya et al. 2005).

McCabe et al. (2005) examined the prevalence rates and correlates of nonprescription use of stimulants (Ritalin, Adderall, or Dexedrine) among US college students and found evidence that misuse is more prevalent among particular subgroups of US college students and types of colleges. The lifetime prevalence of nonprescription stimulant use was 6.9%, past-year prevalence was 4.1%, and past-month prevalence was 2.1%. Multivariate analysis indicated that nonprescription use was higher among college students who were male, white, members of fraternities and sororities and earned lower grade point averages. Wilens et al. (2008) reported similar findings. Rates were higher at colleges located in the northeastern region of the United States and colleges with more competitive admission standards. Nonprescription stimulant users were more likely to report use of alcohol, cigarettes, marijuana, ecstasy, cocaine, and other risky behaviors. Among college students, available evidence suggests that individuals who misuse MPH were more likely to be white, male, affiliated with a formally organized fraternity, and more likely to use other illicit and illegal substances (Bogle and Smith 2009).

A descriptive, nonexperimental, cross-sectional study examined the nonprescription use of stimulants among student pharmacists (Lord et al. 2003). Lifetime prevalence of stimulant misuse was 7% and was more likely in students who were white, older, and fraternity or sorority members, whereas past-year misuse was more likely in whites and low academic achievers. A recent survey found that the misuse of prescription stimulants is also rampant among dental and dental hygiene students (McNiel et al. 2011). The survey, which was mailed to dental education institutions in the south-central region of the United States, found that 12.4% of these students used a stimulant without a prescription and, of those, 70% took it to improve attention and/or concentration. The most commonly reported stimulant medication used was Adderall (77%). The majority (87%) of the students obtained the medication through friends, and 90% began using the drug in college. Interestingly, 17% of the students surveyed felt it was easy to obtain stimulant medication for use at their school, and 17% thought it was a problem within their institution. The use, misuse, and diversion of prescription stimulants among middle and high school students were also examined by McCabe et al. (2005). In this study, the odds for nonprescription stimulant use were lower among African American students and higher among those students with no plans for attending college. These students also had the highest rates of alcohol and other drug use.

The prevalence of prescription stimulant misuse in medical students is also high. In fact, discussion based websites such as Facebook, Medical School Forum, and The Student Doctor Network are rife with Adderall “experts” and informal question-and-answer sessions on the drug. An anonymous survey was administered to 388 medical students (84.0% return rate) across all 4 years of education at a public medical college. More than 10% of medical students reported using stimulants to improve academic performance. ADHD was diagnosed in 5.5% of students and 72.2% of those students were diagnosed after the age of 18 years (Tuttle et al. 2010). This study suggests that medical students appear to be a relatively high-risk population for prescription stimulant misuse. Several officials now say the problem is increasing in medical schools (Harris 2009). “During the last few years, the number of requests for ADD evaluations has hugely increased,” Paula Stoessel, Ph.D., director of mental health services for physicians in training at the University of California, Los Angeles, David Geffen School of Medicine. “We make them [medical students] go through a lot before we hand out medication, but I've heard them talk about [obtaining Adderall prescriptions] in passing.” Clearly, the results emphasize the need for education about stimulants and their adverse side effects.

## Why are prescription stimulants misused?

The reasons why prescription stimulants are misused are numerous and include achieving euphoria, and helping cope with stressful factors related to their educational environment. According to a survey of 334 ADHD-diagnosed college students taking prescription stimulants, 25% misused their own prescription medications to get “high” (Upadhyaya et al. 2005). Like cocaine, MPH inhibits the DAT, which increases synaptic levels of DA, and this is presumed to mediate MPH's reinforcing effects and abuse potential. In laboratory studies, it has been shown that animals will repeatedly administer MPH as they do cocaine (Kollins 2003), and humans receiving both drugs indicate a similar “high” (Volkow et al. 1995). A frequent concern regarding the use of stimulants for ADHD is their mechanism of action, which increases DA and thus may increase the risk for overt, illicit drug use. However, research points to the conclusion that people of any age receiving a stimulant for ADHD have no greater risk for illicit substance abuse compared with the general population (Wilens 2003).

Stimulants are especially popular at the end of a school term when students will often use the drugs to stay awake through the night to study for exams or complete academic projects. In fact, prescription stimulants are most commonly misused to enhance school performance. According to a Web survey of 115 ADHD-diagnosed college students, enhancing the ability to study outside of class was the primary motive for misuse (Rabiner et al. 2009). Pressures such as a persistent desire to succeed academically, poor sleep habits due to large workloads, and the persistence of underlying social and financial demands may place students at an increased risk for misuse of various drugs, including stimulants (Kadison 2005; Teter et al. 2005). Students who misused ADHD medications generally felt that doing so was helpful. Thus, prescription stimulants developed to help children with ADHD improve their focus and attention are often misused by the patient, especially ADHD patients with conduct disorder or comorbid substance abuse (Kollins 2008). Moreover, students without ADHD misuse stimulants to improve performance or to induce euphoria. A web-based survey administered to medical and health profession students found that the most common reason for nonprescription stimulant use was to focus and concentrate during studying (93.5%) (Herman et al. 2011). In this study, approximately 10.4% of students surveyed (45.2% female; 83.9% male; 83.9% Caucasian) have either used a stimulant or are currently using prescription stimulants, and the most commonly abused stimulant (71.4%) was d-AMP. A recent survey found that 70% of dental and dental hygiene students used a prescription stimulant nonmedically to improve attention and/or concentration (McNiel et al. 2011). Student pharmacists (Lord et al. 2003) and medical students (Tuttle et al. 2010) are also using stimulants to improve concentration and academic performance.

## Effects of prescription stimulants on cognition in ADHD

Neuropsychological studies of ADHD children and adults indicate impairments in many cognitive areas including selective attention, memory, reaction time, information processing speed, and executive control function such as set-shifting, and working memory. The benefits of prescription stimulants for enhancing classroom manageability and increasing attention and academic productivity in children are well established. Prescription stimulants may increase the quality of note taking, scores on quizzes and worksheets, writing output, and homework completion. Nevertheless, they do not normalize the ability to learn and apply knowledge (Advokat 2010). In fact, it has been recognized over 30 years that there is little evidence that prescription stimulants such as MPH and AMP improve the academic achievement of ADHD-diagnosed children. Children with ADHD have a consistently lower full-scale IQ than normal controls. They score significantly lower on reading and arithmetic tests, use more remedial academic services, and are more likely to be placed in a special education class, or repeat a grade compared with controls. They also take more years to complete high school and have lower rates of college attendance (Advokat 2010). Thus, prescription stimulants have only a modest impact on these outcomes.

The first review to describe the general academic functioning of adults with ADHD summarized the results from 23 studies (Weyandt and DuPaul 2006). ADHD-diagnosed college students were found to have significantly lower grade point averages, report more “academic problems” and to be less likely to graduate from college. Nevertheless, ADHD-diagnosed college students did not differ in IQ from those without ADHD, and were shown to be able to meet the demands of college courses. On psychological tests, they showed significant deficits in attention, but were not different from normal students on other measures, such as the ability to be flexible and to maintain performance, as task demands varied (Weyandt and DuPaul 2006). More recent reports have reached similar conclusions. Interestingly, like elementary and high school students, college students with ADHD are less likely to reach the same academic level as their non-ADHD counterparts, even when they use stimulant medications. Thus, stimulant medications do not necessarily equalize academic achievement in the typical adult with ADHD.

A recent controlled, cross-sectional study evaluated the effects of stimulants on cognition in adults with ADHD and found that treated ADHD subjects had significantly better scores on measures of IQ than did untreated patients (Biederman et al. 2012). Thus, either good cognitive functioning may be a determinant of seeking treatment or stimulant treatment may improve cognition in adults with ADHD. When ADHD studies address the issue of cognition, they usually demonstrate that treated patients perform better than untreated patients on neuropsychological tests or measures after they are treated. Whether treatment normalizes neurocognitive performance is rarely addressed. In fact, adults with ADHD are less likely to attain the same educational levels as those without the diagnosis relative to what would be predicted based on their IQ, and this outcome does not appear to be improved by stimulant medication. In one recent study, for example, although 84% of ADHD-diagnosed adults were statistically expected to be college graduates, only 50% reached this level of education (Biederman et al. 2008a,b). Gualtieri and Johnson (2008) conducted a cross-sectional study of ADHD patients treated with different ADHD drugs (Adderall XR, atomoxetine, Concerta) (Adderall XR is an extended-release formulation with duration of action of approximately 10–12 h. This is significantly longer than the duration of action of most methylphenidate formulations, with the exception of Concerta. Immediate-release methylphenidate lasts at most for 6 h). Patients' performance on a computerized neurocognitive screening battery was compared with untreated ADHD patients and normal controls. Significant differences were detected between normal and untreated ADHD patients. Treated patients performed better than untreated patients but remained significantly impaired compared with normal subjects. Thus, even after optimal treatment, neurocognitive impairments persisted in the ADHD patients.

It has never been established that the cognitive effects of stimulant drugs are central to their therapeutic utility. In fact, although ADHD medications are effective for the behavioral components of the disorder, little information exists concerning their effects on cognition. Barkley and Cunningham (1978) summarized 17 short-term research studies ranging from 2 weeks to 6 months, and found stimulant medications produced little improvement in the academic performance of hyperkinetic ADHD children. The drugs appeared to reduce disruptive behavior rather than improve academic performance. Stimulant drugs do improve the ability (even without ADHD) to focus and pay attention. One function, which is reliably improved by stimulant medications, is sustained attention, or vigilance. Stimulants improve sustained, focused attention, but “selective attention” and “distractibility” may be worsened, possibly because of a drug induced increase in impulsivity. Both AMP and MPH do not improve (and may even impair) short-term acquisition of information. In addition, AMP and MPH do not improve, and may impair “cognitive flexibility” as assessed with tests such as the Wisconsin Card Sort and Attentional Set-Shifting tasks. MPH has been shown to improve performance on an auditory arithmetic task, the Paced Auditory Serial Addition Task, in adults with ADHD relative to control subjects (Schweitzer et al. 2004). AMP and MPH might improve long-term retention of information, if the drugs are active during a period in which memory is being “consolidated.” However, this may only occur in situations where retention is already suboptimal.

## Effects of stimulants on cognition in individuals without ADHD

Recognition that ADHD persists into adulthood has substantially increased the prescription stimulant treatment of adults with the disorder (see above). It has also resulted in a corresponding escalation of nonprescription stimulant use in many college students confirmed by numerous surveys. Studies consistently show that students report using stimulant medications, legally or illicitly, to improve academic performance, specifically to increase concentration and the ability to stay up longer and study. Intuitively, it would seem logical that drugs that improve attention and concentration should also promote learning and academic achievement. Inherent in terms like “cognitive enhancers,” “smart drugs,” and “neuroenhancers” is the assumption that MPH and d-AMP enhance cognition. Major magazines such as *The New Yorker* have reported a trend toward growing use of prescription stimulants by college students for “neuroenhancement”. In fact, some students are faking ADHD to gain access to prescription stimulant medication, which has led to a shortage of ADHD drugs such as Adderall (Mitchell 2012). Unfortunately, media reports appear to condone this behavior as 95% of articles mentioned at least one possible benefit of using prescription drugs for neuroenhancement, but only 58% mentioned any risks or side effects (Partridge et al. 2011). Duke University recently enacted a new policy prohibiting the nonmedical use of prescription stimulants for any academic purposes (McLaughlin 2012). Students received an email stating policy changes including, “The unauthorized use of prescription medication to enhance academic performance has been added to the definition of Cheating.” In the past, the use of such drugs without a prescription was only a violation under the University's drug policy. Oddly, the assumption that prescription stimulants are truly “cognitive enhancers” is not really questioned. Stimulants reduce hyperactivity, impulsivity, and inattention in children and adults with ADHD, so it has been assumed that these drugs enhance long-term intellectual performance. However, contrary to simple implicit assumptions found in bioethics and media discourses, there are actually only a few studies on the enhancement effects of “cognitive enhancers” in individuals without ADHD.

Smith and Farah (2011) reviewed data on prescription stimulants as neuroenhancers from over forty laboratory studies involving healthy, nonelderly adults. Most of the studies looked at one of three types of cognition: learning, working memory, and cognitive control. Effects of d-AMP or MPH on cognition were assessed by a variety of tasks (Table 1). A typical learning task asks subjects to memorize a list of paired words; an hour, a few days, or a week later, subjects are presented with the first words in the pairs and asked to come up with the second. In general, with single exposures of verbal material, the studies on learning showed that no benefits are seen immediately following learning, but later recall and recognition are enhanced. Of the six articles reporting on memory performance (Rapoport et al. 1978; Soetens et al. 1993; Camp-Bruno and Herting 1994; Fleming et al. 1995; Unrug et al. 1997; Zeeuws and Soetens 2007), encompassing eight separate experiments, only one of the experiments yielded significant memory enhancement on short delays (Rapoport et al. 1978). In contrast, retention was reliably enhanced by d-AMP when subjects were tested after longer delays, with recall improved after 1 h through 1 week (Soetens et al. 1993, 1995; Zeeuws and Soetens 2007). These data suggest that when people are given rote-learning tasks their performance is improved by stimulants. The benefits were more apparent in studies where subjects had been asked to remember information for several days or longer. However, studies only found a correlation with rote memory tasks, not complex memory, which is more likely to appear on college exams.

**Table 1:** Overview of effects of prescription stimulants on cognitive performance in adults without ADHD

Study	Tests	Finding
Barch and Carter (2005)	Spatial working memory	Decrease in reaction time
	Stroop test	Decrease in response time
Breitenstein et al. (2004)	Probabilistic learning	Steeper increase in hits and decrease in misses across learning sessions; increase in retention after more than 1 year
Breitenstein et al. (2006)	Probabilistic learning	Steeper learning curve
Brignell et al. (2007)	Single-exposure verbal learning	At 1 week improved recognition
Brumaghim and Klormart (1998)	Associative learning: word pairs	No effect
Burns et al. (1967)	Associative learning: location of stimulus and response	Slower rate of learning
Callaway (1983)	Item recognition	No effect
Camp-Bruno and Herting (1994)	Repeated-exposure verbal learning	1 h: no effect; 2 h: borderline effect
Camp-Bruno and Herting (1994)	Single-exposure verbal learning	Up to 2.5 h: no effect
Clatworthy et al. (2009)	Spatial working memory	No effect
	Reversal learning	No effect
Cooper et al. (2005)	Continuous performance test (double version)	5 min: decrease in reaction time; decrease in errors of omission
de Wit et al. (2000)	Stop-signal task	No effect
de Wit et al. (2002)	Repeated-exposure verbal learning	25 min: no effect
	Digit span	Increase in performance
	Go/no-go	Decrease in number of false alarms
	Delay of gratification	No effect
Dodds et al. (2008)	Reversal learning	No effect
Elliott et al. (1997)	Spatial span	Decrease in errors
	Spatial working memory	Decrease in errors
	Attentional set-shifting	No effect
	Verbal fluency	No effect
	Sequence generation	No effect
	New Tower of London	No effect
	Tower of London	Relative decrease in accuracy
Fillmore et al. (2005)	Stop-signal task	No effect
	*n*-back	Increase in processing rate
Fitzpatrick et al. (1988)	Item recognition: stimulus evaluation/response selection task	Decrease in reaction time
Fleming et al. (1995)	Single-exposure verbal learning	20 min: no effect on single-exposure verbal learning
	Continuous performance test	5 min: decrease in reaction time
	Spatial working memory	No effect
	Wisconsin Card Sorting Test	No effect
	Verbal fluency	No effect
Hurst et al. (1969)	Associative learning: word pairs	Increase in retention after 1 week delay
Kennedy et al. (1990)	Item recognition	No effect
	Grammatical reasoning	No effect
Klorman et al. (1984)	Continuous performance test (BX version)	45 min: decrease in reaction time; 12.5/45 min: decrease in errors of omission
Koelega (1993)	Vigilance performance	Improves the overall level of vigilance performance and prevents the decrement that occurs over time under normal circumstances
Kumari et al. (1997)	Motor sequence learning	No effect
Makris et al. (2007)	Motor sequence learning	No effect
	Item recognition	Proportion correct sustained across multiple trials
Mattay et al. (1996)	Wisconsin Card Sorting Test	No effect
Mattay et al. (2000)	*n*-back	No effect
Mattay et al. (2003)	*n*-back	No effect
	Wisconsin Card Sorting Test	No effect
Mehta et al. (2000)	Spatial working memory	Decrease in between-search errors
Mintzer and Griffiths (2007)	Single-exposure verbal learning	2 h: improved recognition; no effect on recall
	*n*-back	No effect
	Item recognition	No effect
Oken et al. (1995)	Digit span	No effect
Rapoport et al. (1978)	Single-exposure verbal learning; continuous performance test (BX version)	10 min: improved recall; decrease in errors of omission
Rogers et al. (1999)	Attentional set-shifting	Increase in intradimensional shift errors; decrease in extradimensional shift errors; increase in response latencies
Schmedje et al. (1988)	Digit span	No effect
	Pattern memory	No effect
Schroeder et al. (1987)	Strategic choice task	Decrease in changeover rate
Servan-Schreiber et al. (1998)	Flanker task	Decrease in response time; increase in accuracy
Silber et al. (2006)	Digit span	No effect
	Trail Making Test	No effect
Soetens et al. (1993)	Single-exposure verbal learning	20 min: no effect; 1 h–3 days: improved long-term retention
Soetens et al. (1995)	Single-exposure verbal learning	1 h–1 week: improved long-term retention in free recall; 1 week: improved recognition
Strauss et al. (1984)	Associative learning: word pairs; continuous performance test (double version)	No effect; 45 min: decrease in reaction time; 45 min: decrease in errors of omission
Unrug et al. (1997)	Single-exposure verbal learning	20 min: no effect
Ward et al. (1997)	Motor sequence learning; item recognition	No effect; decrease in reaction time
Weitzner (1965)	Associative learning: word pairs	Improved performance only when pairs were uniquely semantically related
Willett (1962)	Repeated-exposure verbal learning	Decrease in number of trials to reach criterion
Zeeuws and Soetens (2007)	Single-exposure verbal learning	30 min: no effect; 1 h–1 day: improved long-term retention

Table adapted with permission from Smith and Farah (2011), Copyright 2011 by the American Psychological Association. The use of APA information does not imply endorsement by APA.

In contrast to the types of memory, which are long lasting and formed as a result of learning, working memory is a temporary store of information that plays a role in executive function. Several studies have assessed the effect of MPH or d-AMP on tasks examining various aspects of working memory (Sahakian and Owen 1992; Oken et al. 1995; Elliott et al. 1997; Mehta et al. 2000; Barch and Carter 2005; Silber et al. 2006; Clatworthy et al. 2009) (see Table 1). One classic approach to the assessment of working memory is the span task, in which a series of items is presented to the subject for repetition, transcription, or recognition. A spatial span task, in which the subjects must retain and reproduce the order in which boxes in a scattered spatial arrangement change color was employed by Elliott et al. (1997) to assess the effects of MPH on working memory. For the subjects in the group who received placebo first, MPH increased spatial span. However, for the subjects who received MPH first, there was a nonsignificant opposite trend. The authors noted that the subjects in the first group performed at an overall lower level, and so, this may have contributed to the larger enhancement effect for less able subjects. Barch and Carter (2005) obtained similar results and Mehta et al. (2000) found evidence of greater accuracy with MPH. In the study by Mehta et al. (2000), the effect depended on subjects' working memory ability: the lower a subject's score on placebo, the greater the improvement on MPH. In contrast to the three previous studies, Bray et al. (2004) reported that MPH does not improve the cognitive function of sleep-deprived young adults. In sum, the evidence concerning stimulant effects of working memory is mixed, with some findings of enhancement and some null results, although no findings of overall performance impairment (Smith and Farah 2011). However, the small effects were mainly evident in subjects who had low cognitive performance to start with, showing that the drug is more effective at correcting deficits than “enhancing performance.” Farah et al. (2009) recently examined the effect of Adderall upon creativity, a component of cognition stimulants are suspected of stifling, in a double-blind, placebo-controlled trial. They found that the drug enhanced creativity on specific tasks, but the amount of enhancement depended upon the baseline performance of individuals: lower-performing individuals were more enhanced than high-performers. Thus, the drugs do not offer as much help to people with greater intellectual abilities.

The third type of cognition is cognitive control. Cognitive control is a broad concept that refers to guidance of cognitive processes in situations where the most natural, automatic, or available action is not necessarily the correct one (Smith and Farah 2011). Attention and working memory are thought to rely on cognitive control and loss of cognitive control is a major component of many neuropsychiatric diseases such as schizophrenia. The effects of MPH and d-AMP have been determined on several tests used to study cognitive control, including the go/no-go task, the stop-signal task, and the Flanker test. In general, the effects of stimulants on cognitive control are not robust, but MPH and d-AMP appear to enhance cognitive control in some tasks for some people, especially those less likely to perform well on cognitive control tasks (Smith and Farah 2011). The results of these studies currently provide limited support for the enthusiastic portrayals of cognitive enhancement.

The neural basis of error processing has become a key research interest in cognitive neuroscience. Recently, a single dose of MPH was shown to improve the ability of healthy volunteers to consciously detect performance errors (Hester et al. 2012). Furthermore, this behavioral effect was associated with a strengthening of activation differences in the dorsal anterior cingulate cortex and inferior parietal lobe during the MPH condition for errors made with versus without awareness. How the brain monitors ongoing behavior for performance errors is a central question of cognitive neuroscience. Diminished awareness of performance errors limits the extent to which humans engage in corrective behavior and has been linked to loss of insight in ADHD and drug addiction.

As it remains unclear whether stimulant medication has the same effect on healthy individuals as for those with ADHD, it is possible that many reported effects of prescription stimulants in healthy individuals may stem from placebo effects. Looby and Earleywine (2011) examined whether placebo effects influence reports of subjective mood and cognitive performance among college students who endorsed several risk factors for prescription stimulant misuse (e.g., low grade point average, fraternity/sorority involvement, binge drinking). Interestingly, participants believed that they had better ability to focus and persevere, particularly for a sustained amount of time, when they expected to receive MPH (Looby and Earleywine 2011). This is similar to circumstances in which participants may engage in nonmedical-stimulant use to study or cram for extended hours. On the other hand, when experimental participants did not expect to receive MPH, their attention appeared disrupted resulting in inconsistent reaction times throughout the CPT. Interestingly, subjective feelings of being high and stimulated were produced solely by expecting to receive MPH. This finding is important to consider when examining initiation and maintenance of nonmedical prescription stimulant use. As motives for nonprescription stimulant use include the desire to feel high (Barrett et al. 2005), it is likely that individuals who use a stimulant for this purpose will consequently feel high due to these demonstrated placebo effects, which will likely maintain misuse of the drug.

## Prescription stimulant misuse in athletes

ADHD is a controversial problem in sport as participants with this disorder often require banned stimulants while competing. Many of the governing bodies of competitive sports have developed regulations that limit the use of stimulant medications to treat ADHD. In other cases, stimulant use is allowed in the setting of a documented diagnosis of ADHD. Most sports organizations around the world now follow the guidelines set forth by the World Anti-Doping Agency (WADA). According to this document, the diagnosis of ADHD is to be made by “experienced clinicians” and in accordance to the DSM-IV. Stimulant medications are considered to be a “medical best practice treatment” that do require the athlete to file a therapeutic use exemption (TUE). A TUE gives athletes with medical diagnoses an exemption to use a drug normally prohibited by MLB, to treat a legitimately diagnosed medical condition. WADA recommends reassessments of continued treatment every 3–4 months. Other organizations, such as the National College Athletic Association (NCAA) and individual professional leagues, such as the National Football League (NFL) and Major League Baseball (MLB), have developed their own regulations.

The NCAA does not require that physicians prescribe a trial of nonstimulant medications before prescribing stimulants, only that the prescribing physician considers nonstimulants first. The NCAA acknowledges that nonstimulant medication may not be as effective as stimulant medications in treating ADHD. In contrast to the NCAA regulations, athletes who are also participating in events governed by the International Olympic Committee (IOC) and/or WADA are not allowed to use stimulant medications, even with a TUE. These organizations require that the athlete with ADHD on stimulant medications stop taking these medication or risk disqualification (Putukian et al. 2011).

It has been reported that MLB players are using an ADHD diagnosis to evade the AMP ban (Associated Press 2009). According to records MLB officials turned over to congressional investigators as part of George Mitchell's probe into steroid use in baseball, the number of players getting “therapeutic use exemptions” from baseball's AMP ban jumped in 1 year from 28 to 103 – which means that, suddenly, 7.6% of the 1354 players on major-league rosters have been diagnosed with ADHD. MLB banned AMP in 2006. The prevalence of ADHD in athletes has not been studied, although there is no reason to believe it would differ from the general population. Thus, 2–3 times the usual adult rate of ADHD in baseball players is alarming. Athletes may see stimulants as a way to help maintain physical fitness for their competitive sport or to improve their concentration. Certainly some of the players getting prescriptions for ADHD medications may have a legitimate medical need and without treatment, players manifesting the symptoms of untreated ADHD would be at a disadvantage to non-ADHD players. A therapeutic dose of MPH will benefit concentration, and may improve motor coordination. Prescription stimulants to treat ADHD could be used as performance enhancing drugs (PEDs); however, a proper diagnosis would prevent athletes from abusing the TUE status to “cheat within the rules.”

Some athletes will only take medications episodically for school testing or for studying purposes. Others may feel that their sport performance is improved on stimulants, whereas others may temporarily stop taking them so that their sports play is more random and unfocused, which they feel improves their performance (Pelham et al. 1990).

## Potential adverse affects of chronic stimulant use

ADHD is now recognized as a chronic disorder that continues into adulthood; therefore, some individuals take stimulants such as MPH and d-AMP for years. The medical literature provides abundant data to support the potentially positive effect of stimulants for the majority of children, adolescents, and adults with ADHD, and stimulants have been considered to be relatively safe (Elia et al. 1999; Brown et al. 2005). However, reports of adverse events in conjunction with the use of these drugs have raised concern about their safety.

Large doses of stimulants can lead to psychosis, seizures, and cardiovascular events. The induction of schizophrenic-like states in AMP abusers is well documented, although the onset of such states in children on prescribed doses of stimulant medication is observed far less often (Polchert and Morse 1985; Masand et al. 1991; Murray 1998). Surles et al. (2002) published a case report of psychotic reactions to AMP (10 mg/day) in an adolescent ADHD patient. The patient displayed many of the characteristics of AMP-induced psychosis including visual hallucinations, delusions, anorexia, flattening of affect, and insomnia. It is thought that the mechanism of AMP-induced psychosis is mediated by dopaminergic excess. As the patient's symptoms disappeared when taken off the stimulant medication, it suggests that the psychosis was indeed secondary to AMP.

The most commonly observed cardiovascular effects linked with ADHD stimulant medications include hypertension and tachycardia. In addition, cardiomyopathy, cardiac dysrhythmias, and necrotizing vasculitis have been described. In February 2005, the brand medication Adderall XR (Shire BioChem Inc, Quebec, Canada) was withdrawn from the Canadian market by Health Canada. Case reports on serious cardiovascular adverse drug reactions (ADRs), sudden death, and psychiatric disorders led regulatory agencies to warn against the use of MPH in the pediatric population in 2006 and 2007 (European Medicines Agency 2007). In 2006, warnings were also linked to atomoxetine use due to reports of hepatotoxicity and suicidal thoughts in children. These concerns received glaring attention in 2006 and led the US Food and Drug Administration advisory committee to propose placing a black box warning concerning sudden death on psychostimulants in response to ADR reports.

Adderall use is associated with myocardial infarction and even sudden death (Gandhi et al. 2005; Jiao et al. 2009). Gandhi et al. (2005) reported the case of a 15-year-old male subject who suffered a myocardial infarction after taking two 20 mg tablets of Adderall. Jiao et al. (2009) reported a second case of a 20-year-old ADHD college freshman with myocardial infarction after taking two 15-mg tablets of Adderall XR. Recently, Sylvester and Agarwala (2012) reported another case of a 15-year-old male subject who suffered a myocardial infarction after starting Adderall XR. The patient was otherwise in good health with no previous cardiac abnormalities and improved with cessation of medication. The findings of the case have been disputed (Rosenthal 2012).

In addition, a recent report by Alsidawi et al. (2011) discusses the case of a 19-year-old female subject with Adderall overdose induced inverted-Takotsubo cardiomyopathy, also known as stress-induced cardiomyopathy. The patient was brought to the emergency department after ingesting 30 Adderall tablets. She complained of pressure like chest pain and shortness of breath. Her cardiac enzymes were elevated, but the electrocardiogram was unremarkable. Echocardiography identified a low ejection fraction of 25–35% with severe hyperkinetic apex and akinetic base consistent with the diagnosis of inverted-TTC. Her symptoms resolved in 24 h. Drug-induced-Takotsubo cardiomyopathy has been previously reported and is mainly attributed to sympathetic overstimulation (Amariles 2011). In this case, the patient overdosed on Adderall, which is a sympathomimetic drug. The mechanisms for AMP-induced cardiac injury are postulated to be similar to those seen with cocaine, which include coronary spasm, prothrombotic state, accelerated atherosclerosis due to endothelial injury, and direct myocardial (Chen 2007). Inappropriate dosing or taking with alcohol increases the risk of serious cardiovascular side effects like myocardial infarction, even without underlying cardiovascular risk factors.

Unfortunately, there are few long-term studies (i.e., longer than 24 months) on the use of stimulants for the management of ADHD; therefore, the precise long-term effects – either adverse or positive – remain unknown. A recent study (Vitiello et al. 2011) suggests that the chronic use of stimulant medication to treat ADHD in children does not appear to increase the risk for high blood pressure in the long term, but it may have modest effects on heart rate. The MTA study found that stimulant medication does not appear to increase the risk for abnormal elevations in blood pressure or heart rate over a 10-year period; however, the effect of stimulants on heart rate can be detected even after years of use (Vitiello et al. 2011). The effect on heart rate may be clinically significant for individuals who have underlying heart conditions.

A cohort study sought to determine whether use of MPH in adults is associated with elevated rates of serious cardiovascular events compared with rates in nonusers (Schelleman et al. 2012). All new MPH users with at least 180 days of prior enrollment were identified. Initiation of MPH was associated with a 1.8-fold increase in risk of sudden death or ventricular arrhythmia; however, the lack of a dose response relationship suggested that this association might not be a causal one. A recent study by Habel and colleagues (Habel et al. 2011), which compared approximately 150,000 adults prescribed ADHD medication with approximately 300,000 nonusers, found no evidence of a link between ADHD medication and cardiovascular risk (myocardial infarction, sudden death, or stroke). Although the student enrolled adults, the same group also has reported a similar lack of significant association between serious cardiovascular events and use of ADHD medications in children and younger adults (Cooper et al. 2011). These findings support the final decision of the US Food and Drug Administration committee to not to place a black box warning for all children and adults, but to pursue further research. However, the study by Habel et al. (2011) has limitations stemming from its focus on the most severe cardiovascular event. The databases were not used to examine other cardiovascular adverse effects, such as palpitations and dyspnea, which, although less severe, are nonetheless alarming to patients.

Additional potential ADRs associated with stimulant use are important to note including abdominal pain, anorexia, constipation, dizziness, dry mouth, headache, insomnia, jitteriness, irritability, nausea, and palpitations (Greydanus and Strasburger 2006). College students with ADHD who misuse prescribed stimulants also reported hyperactivity symptoms as a common adverse event. Of particular significance to athletes, many stimulants utilized in treating ADHD may increase core temperature (Piper et al. 2005), possibly increasing risk of heart injury. These agents may also mask signs and symptoms of fatigue and allow for a longer duration of exercise with elevated temperature in excess of 40°C. Thus, in situations of increased exogenous heat stress, stimulants should be used with caution.

## Conclusion

Although prescription stimulants have been shown to be relatively safe and effective in managing the symptoms of ADHD, there exists a significant potential for misuse. The data are clear that individuals with and without ADHD, including athletes misuse stimulants to enhance performance. Although stimulants may improve an individual's performance when given a rote-learning task, they do not offer as much help to people with greater intellectual abilities. Stimulants do not increase IQ (Advokat et al. 2008). In fact, very little is known about the effects of nonprescription stimulants on cognitive enhancement outside of the student population, although it is frequently reported in newspaper articles. Thus, the rumored effects of “smart drugs” may be a false promise, as research suggests that stimulants are more effective at correcting deficits than “enhancing performance.” Moreover, students are taking unnecessary risks including the potential for harmful side effects, which may cause sudden death. This requires education on the proper use of stimulants and on the signs and symptoms of misuse and the health risks associated with misuse. It is important that students with prescription stimulants understand that they are the main source of diversion to other students, and should receive education in the prevention of stimulant diversion. Health centers should aim to recognize students who are misusing stimulants because they may present with a variety of signs including insisting on a larger dose, and demanding more drug during times within the academic year, such as during finals. Students with past or active drug abuse patterns should not be prescribed stimulants, as they are more likely to divert their prescription stimulants. It is also important that athletes be warned that the NCAA, the US Olympic Committee, and the IOC ban MPH. As a result, education on the proper use of stimulants and on the signs and symptoms of misuse is an imperative.
